# Reliability of P3 Event-Related Potential During Working Memory Across the Spectrum of Cognitive Aging

**DOI:** 10.3389/fnagi.2020.566391

**Published:** 2020-10-19

**Authors:** Hannes Devos, Jeffrey M. Burns, Ke Liao, Pedram Ahmadnezhad, Jonathan D. Mahnken, William M. Brooks, Kathleen Gustafson

**Affiliations:** ^1^Laboratory for Advanced Rehabilitation Research in Simulation, Department of Physical Therapy and Rehabilitation Science, University of Kansas Medical Center, Kansas City, KS, United States; ^2^Department of Neurology, University of Kansas Medical Center, Kansas City, KS, United States; ^3^University of Kansas Alzheimer’s Disease Center, University of Kansas Medical Center, Kansas City, KS, United States; ^4^Hoglund Brain Imaging Center, University of Kansas Medical Center, Kansas City, KS, United States; ^5^Department of Biostatistics & Data Science, University of Kansas Medical Center, Kansas City, KS, United States

**Keywords:** event-related potentials, electro-encephalography, reliability, working memory, older adults, mild cognitive impairment, Alzheimer’s disease, pre-clinical AD

## Abstract

Event-related potentials (ERPs) offer unparalleled temporal resolution in tracing distinct electrophysiological processes related to normal and pathological cognitive aging. The stability of ERPs in older individuals with a vast range of cognitive ability has not been established. In this test-retest reliability study, 39 older individuals (age 74.10 (5.4) years; 23 (59%) women; 15 non β-amyloid elevated, 16 β-amyloid elevated, 8 cognitively impaired) with scores on the Montreal Cognitive Assessment (MOCA) ranging between 3 and 30 completed a working memory (*n*-back) test with three levels of difficulty at baseline and 2-week follow-up. The main aim was to evaluate stability of the ERP on grand averaged task effects for both visits in the total sample (*n* = 39). Secondary aims were to evaluate the effect of age, group (non β-amyloid elevated; β-amyloid elevated, cognitively impaired), cognitive status (MOCA), and task difficulty on ERP reliability. P3 peak amplitude and latency were measured in predetermined channels. P3 peak amplitude at Fz, our main outcome variable, showed excellent reliability in 0-back (intraclass correlation coefficient (ICC), 95% confidence interval = 0.82 (0.67–0.90) and 1-back (ICC = 0.87 (0.76–0.93), however, only fair reliability in 2-back (ICC = 0.53 (0.09–0.75). Reliability of P3 peak latencies was substantially lower, with ICCs ranging between 0.17 for 2-back and 0.54 for 0-back. Generalized linear mixed models showed no confounding effect of age, group, or task difficulty on stability of P3 amplitude and latency of Fz. By contrast, MOCA scores tended to negatively correlate with P3 amplitude of Fz (*p* = 0.07). We conclude that P3 peak amplitude, and to lesser extent P3 peak latency, provide a stable measure of electrophysiological processes in older individuals.

## Introduction

The aging process is characterized by gradual decline in physical, neurobiological and cognitive functions that may impact instrumental activities of daily living (iADL) such as driving, doing household chores, managing finances, medication adherence, or grocery shopping (Moon et al., 2018; Carmona-Torres et al., 2019). Deterioration in these iADL becomes more apparent with age-related neurodegeneration such as mild cognitive impairment (MCI) and Alzheimer’s disease (AD) (Jekel et al., 2015). Executive functions in particular are paramount in carrying out numerous iADL, but are also vulnerable to the effects of normal and pathological cognitive aging (Overdorp et al., 2016; Tabira et al., 2020). Working memory is one core executive function that relates to the ability to temporarily store, process, and manipulate the information necessary for higher order cognitive tasks such as decision making, learning, and reasoning (Baddeley, 1992). Working memory stems from the interaction between attention, short-term retention and manipulation of information, carried out by the coordinated activation of many brain regions (Eriksson et al., 2015).

The prefrontal cortex has particularly been associated with working memory (Bahmani et al., 2019). Consequently, the prefrontal cortex is highly susceptible to the effects of aging and early neurodegeneration (West et al., 2002; Ranchet et al., 2017). A recent meta-analysis pooling functional magnetic resonance imaging studies suggested a gradual and linear decline in prefrontal cortex engagement in older individuals (Yaple et al., 2019). Similarly, electrophysiological processes also decline with age. The P3, a positive peak that appears with a latency between 250 to 500 ms in the event-related potential (ERP), has been implicated in attention and working memory processes across the lifespan (Van Dinteren et al., 2014). A previous study showed reduced positivity in P3 central-frontal and parietal ERPs in older adults (Lubitz et al., 2017), whereas others demonstrated frontal hyperactivity in P3 coupled with parietal or posterior hypoactivity (Fjell and Walhovd, 2001; Saliasi et al., 2013). Despite the ambiguity in ERP findings, most studies conclude that the abnormal ERP response in older individuals reflects inefficient or compensatory use of neural resources due to frontal cortex dysfunction (Saliasi et al., 2013; Lubitz et al., 2017). Therefore, electrophysiological responses to working memory tasks are convenient measures to test hypotheses related to frontal cortex function, normal cognitive aging, and early neurodegeneration.

The ability to distinguish natural variability and measurement error from biologically relevant cognitive changes due to aging or early neurodegeneration is valuable to provide informed decisions on diagnosis, monitoring, and treatment of cognitive impairments (Feinkohl et al., 2020). However, older adults show more intraindividual variability in performance measures of working memory compared to younger adults. The age-related changes in intraindividual variability of performance measures become even more apparent with increasing cognitive demand (West et al., 2002). This increased intraindividual variability may also stem from the heterogeneity of cognitive profiles in older individuals, especially when patients with MCI and AD are included (Troyer et al., 2016). The intraindividual variability observed in performance measures is believed to be linked to frontal cortex dysfunction (West et al., 2002), which may therefore also affect intraindividual variability of the ERP response in older adults (Robertson et al., 2006). To date, few studies have investigated test-retest reliability of P3 ERP in healthy older adults (Sandman and Patterson, 2000; Walhovd and Fjell, 2002; Behforuzi et al., 2019). The test-retest reliability of the P3 ERP in older individuals with a heterogeneous cognitive profile has yet to be established.

The main aim was to characterize test-retest reliability of P3 ERP in a group of older adults with a wide range of cognitive function. Secondary aims were to investigate the impact of age, disease groups (non β-amyloid elevated; β-amyloid elevated, cognitively impaired), cognitive status, and task difficulty on P3 ERP.

## Materials and Methods

### Participants

This test-retest reliability study included 39 right-handed participants recruited from the KU Disease Center between 05/03/2018 and 03/10/2020. Inclusion criteria were informed consent; age older than 65; ability to understand the instructions in English; and having previously undergone an amyloid PET scan of the brain. Cerebral amyloid burden was assessed using PET images, obtained on a GE Discovery ST-16 PET/CT scanner after administration of intravenous florbetapir F-18. Standard Uptake Value Ratio for six regions of interest was calculated using MIMneuro software (MiM Software Inc., Cleveland, OH, United States) by normalizing the Aβ PET image to the entire cerebellum to calculate the. Diagnosis of cognitively normal pre-clinical AD followed the recommendations from NIA and the Alzheimer’s Association workgroup (Sperling et al., 2011). The protocol for determination of amyloid elevation is detailed elsewhere (Vidoni et al., 2016). The average time between administration of PET scan and EEG assessment was 1090 (479) days. Exclusion criteria were: currently taking steroids, benzodiazepines, or neuroleptics; history of any substance abuse; and history of a neurological disorder other than MCI or AD. Sixteen were cognitively normal older adults with no elevated amyloid PET scans (Aβ−), 15 were cognitive normal with elevated amyloid PET scans (Aβ+), and eight had a clinical diagnosis of MCI or AD with positive amyloid PET scans. Participants completed their 2-week follow-up session 16 ± 8 days after the first session. Each session lasted about 60 minutes including rest breaks.

### Procedure

#### Demographic and Clinical Information

Age, sex, and education were recorded. General cognitive functions were evaluated with the Montreal Cognitive Assessment (MOCA) (Nasreddine et al., 2005). Scores on the MOCA range between 0 and 30.

#### *N*-Back Test

In the *n*-back test, participants are shown a series of letters and are instructed to press a button when the current stimulus is the same as the item presented *n*-positions back. The cognitive demand of the *n*-back task increases with each number, while the perceptual and motor demands remain constant. In this study, the 0-back, 1-back, and 2-back tests were administered. The 0-back test is essentially a memory search task of sustained attention and often used as a control condition (Miller et al., 2009; Bopp and Verhaeghen, 2018). The 1-back test requires the participant to passively store and update information in working memory. Whereas in the 0-back and 1-back the stimulus on screen is held in the focus of attention, the 2-back test requires constant switching from the focus of attention to short-term memory (Bopp and Verhaeghen, 2018). Higher levels of difficulty require continuous mental effort to update information of new stimuli and maintain representations of recently presented stimuli (Gevins et al., 2011).

Participants sat in a comfortable chair at 26 inches in front of the computer screen with the center of the screen at eye level. White letters appeared on a black screen. Prior to each test, participants were given a practice trial consisting of 7 non-targets and 3 targets. The practice trials were repeated until the participant felt comfortable with the instructions. Each test comprised 180 trials, including 60 trials that needed a response (target, 33.3%) and 120 trials for which a response was not required (non-target, 66.7%). Each letter was presented for 500 ms on the computer screen followed by a blank interstimulus interval for 1,700 ms, with a random jitter of ±50 ms. The maximum time to accept the response was 2,150 ms. The total task time was ∼7 minutes. In the 0-back test, participants were instructed to press the left mouse button as soon as the letter “X” (target) appeared on the screen while ignoring the other letters (non-target). In the 1-back test, participants were instructed to press the button if the current letter on the screen was the same as the letter previously shown (target). In the 2-back test, participants were instructed to press the button when the current letter was the same as the one presented two places before (target). The number of hits (accuracy) and response times to the hits were the main behavioral performance outcome measures.

#### P3 ERP

Continuous electro-encephalogram (EEG) was acquired using a Philips EGI high-density system from 256 scalp electrodes, digitized at 1,000 Hz. Data were filtered from 0.50 to 30 Hz using EGI software. Data were online referenced to Cz and offline rereferenced to the averaged mastoids. All other EEG processing was done in EEGLab (Delorme and Makeig, 2004) and in ERPLab (Lopez-Calderon and Luck, 2014). Various artifacts unrelated to cognitive functions, including ocular and muscular movement or cardiovascular signals, were identified and removed using independent component analysis (ICA). Signals from bad electrodes were interpolated using surrounding electrode data. Stimulus-locked ERPs were extracted from the *n*-back tests and segmented into epochs of 100 ms before to 1,000 ms after stimulus onset, and baseline corrected using the prestimulus interval. Scalp locations and measurement windows for the P3 component were based on their spatial extent and latency after inspection of grand average waveforms (collapsed across the two sessions). P3 peak amplitude of the task effect was considered the main electrophysiological outcome measure, but we also used P3 peak latency as outcome measure. The task effect was calculated by subtracting the average ERP elicited from the targets from the average ERP elicited by non-targets for each participant. The P3 component time window was established between 200 and 400 ms for all three tests. Because of the prefrontal cortex involvement in working memory, we identified *a priori* Fz as the main channel, but also calculated reliability of other pre-identified electrode locations, i.e., Cz, Pz, F3, and F4. Cz was interpolated using the surrounding five channels. No participants were removed from the analyses because of artifacts. However, one participant disengaged during the 2-back test and was therefore excluded from the 2-back reliability analyses.

### Data Analysis

Descriptive analysis including mean (standard deviation) and frequency count of participants’ general, performance measures, and ERP data were performed as appropriate. Intra-class correlation coefficients (ICC) were used to calculate test-retest reliability of performance measures and P3 amplitude and latency. ICCs reflect the consistency of a measure taking into account variance related to the time of testing (Shrout and Fleiss, 1979). ICC values less than 0.40 were considered poor; values between 0.40 and 0.59 fair, values between 0.60 and 0.74 good, and values between 0.75 and 1.00 excellent (Cicchetti, 1994). Bland-Altman plots were used to visualize the measurement precision of amplitude and latency across the test moments (Bland and Altman, 1986). Intersubject stability according to subject rankings was calculated using the Pearson r correlation coefficient. Generalized linear mixed models were employed to evaluate the effect of age, diagnosis (Aβ−; Aβ+; MCI/AD), MOCA scores, and task difficulty on stability of the P3 amplitude and latency. Stability of P3 amplitude (latency) was calculated as the squared difference of P3 amplitude (latency) at follow-up and baseline. The Kolmogorov–Smirnov test was employed to test the normality of our data distribution in addition to visualization of Q-Q plots. All analyses were done using SAS 9.4 software. The threshold of significance was set at *p* = 0.05.

## Results

### Participant Characteristics

Participants (*n* = 39) were on average 74.05 (5.37) years old and scored 26.44 (4.76) on the MOCA scale. MOCA scores ranged between 3 and 30. No differences were observed for age and sex between groups. As expected, participants with MCI/AD scored worse on the MOCA compared to Aβ− and Aβ+ (Table 1).

**TABLE 1:** Participant characteristics of total sample and subgroups.

**Variable**	**Total sample (*n* = 39)**	**Aβ− (*n* = 15)**	**Aβ+ (*n* = 16)**	**MCI/AD (*n* = 8)**	***p*-value**
Age, years	74.05 (5.37)	74.88 (6.15)	72.88 (5.30)	75.00 (3.12)	0.50
Sex, female (%)	23 (60)	9 (60)	11 (69)	3 (38)	0.36
MOCA, score	26.44 (4.76)	28.06 (1.53)	26.69 (2.75)	22.00 (8.80)	0.009

*Abbreviations: Aβ−, beta amyloid non-elevated; Aβ+, beta amyloid elevated; AD, Alzheimer’s disease; MCI, mild cognitive impairment, MOCA, Montreal Cognitive Assessment.*

### Test-Retest Reliability of Performance Measures

All ICC values of hits (accuracy) and response times of each *n*-back test demonstrated excellent reliability (Supplementary Table 1). ICCs of hits ranged between 0.92 (1- and 2-back) and 0.99 (0-back) and were slightly higher than the ICCs of response times, ranging between 0.76 (2-back) and 0.89 (1-back). Pearson r correlations ranged from 0.65 (0-back response time) to 0.99 (0-back hits).

### Test-Retest Reliability of ERP Measures

**FIGURE 1:**
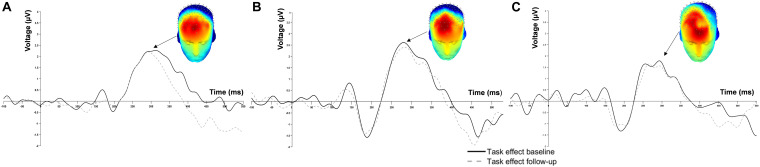
Grand average event-related potential waveform at Fz of **(A)** 0-back, **(B)** 1-back and **(C)** 2-back.

Grand average waveforms of the task effect from all channels at baseline and follow-up are displayed in Figure 1. The 3D scalp map is embedded in the figure to demonstrate the task effect at P3. Considerable overlap in ERP response within the P3 time window (200–400 ms post-stimulus) was observed at baseline and 2-week follow-up.

The ICC values of P3 peak amplitude and peak latency of the key electrode locations are displayed in Table 2. Overall, P3 amplitude showed greater reliability compared to P3 latency across channels and task difficulty levels. Also, ICCs of the 0-back and 1-back were consistently higher than those calculated for the 2-back.

**TABLE 2:** Comparison of task effect (target–non-target) P3 peak response at baseline and 2-week follow-up.

**Variable**	**Baseline**	**Follow-up**	**Pearson *r***	**ICC (95% CI)**
0-back, Fz amplitude (μV)	4.87 (3.23)	5.02 (3.13)	0.63^a^	0.82 (0.67–0.90)^a^
0-back, Fz latency (ms)	291.49 (49.98)	293.31 (40.26)	0.38^b^	0.54 (0.13–0.76)^b^
0-back, Cz amplitude (μV)	2.71 (2.46)	2.59 (2.20)	0.56^a^	0.73 (0.48–0.85)^a^
0-back, Cz latency (ms)	289.49 (55.06)	290.03 (46.57)	0.30^b^	0.51 (0.07–0.74)^a^
0-back, Pz amplitude (μV)	1.94 (1.47)	2.01 (2.00)	0.38^b^	0.54 (0.11–0.76)^b^
0-back, Pz latency (ms)	309.74 (62.36)	315.92 (63.96)	0.34^b^	0.52 (0.08–0.75)^b^
0-back, F3 amplitude (μV)	3.99 (2.80)	4.29 (2.41)	0.58^a^	0.74 (0.49–0.86)^a^
0-back, F3 latency (ms)	297.33 (45.51)	295.51 (38.37)	0.31^b^	0.47 (−0.02 to 0.72)^b^
0-back, F4 amplitude (μV)	4.29 (2.93)	4.44 (2.72)	0.62^a^	0.77 (0.56–0.88)^a^
0-back, F4 latency (ms)	300.54 (52.41)	297.95 (35.36)	0.31	0.45 (−0.05 to 0.71)^b^
1-back, Fz amplitude (μV)	3.97 (3.27)	4.08 (3.76)	0.78^a^	0.87 (0.76–0.93)^a^
1-back, Fz latency (ms)	300.90 (38.45)	300.26 (45.77)	0.31	0.47 (−0.02 to 0.72)^a^
1-back, Cz amplitude (μV)	3.48 (3.05)	3.56 (3.02)	0.79^a^	0.86 (0.74–0.92)^a^
1-back, Cz latency (ms)	302.11 (37.55)	300.03 (42.39)	0.33	0.44 (−0.04 to 0.70)^a^
1-back, Pz amplitude (μV)	1.64 (1.51)	1.54 (1.70)	0.58^a^	0.73 (0.48–0.86)^a^
1-back, Pz latency (ms)	318.44 (66.66)	309.97 (64.17)	0.26	0.42 (−0.11 to 0.69)^b^
1-back, F3 amplitude (μV)	3.30 (3.43)	3.42 (3.47)	0.76^a^	0.87 (0.74–0.93)^a^
1-back, F3 latency (ms)	301.92 (44.80)	300.67 (49.86)	0.38^b^	0.55 (0.14–0.77)^b^
1-back, F4 amplitude (μV)	3.51 (2.95)	3.46 (3.37)	0.68^a^	0.81 (0.64–0.90)^a^
1-back, F4 latency (ms)	304.44 (43.16)	311.03 (45.42)	0.59^a^	0.75 (0.51–0.87)^a^
2-back, Fz amplitude (μV)	3.39 (2.29)	3.21 (2.15)	0.36^b^	0.53 (0.09–0.75)^b^
2-back, Fz latency (ms)	300.97 (39.67)	303.68 (45.65)	0.09	0.17 (−0.60 to 0.49)
2-back, Cz amplitude (μV)	2.28 (1.26)	2.08 (1.65)	0.39^b^	0.51 (0.12–0.78)^b^
2-back, Cz latency (ms)	300.63 (49.62)	302.21 (45.84)	0.31	0.46 (−0.04 to 0.70)
2-back, Pz amplitude (μV)	1.24 (1.20)	1.38 (1.83)	−0.03	−0.06 (−1.04 to 0.44)
2-back, Pz latency (ms)	318.74 (66.70)	321.63 (63.18)	0.08	0.15 (−0.63 to 0.56)
2-back, F3 amplitude (μV)	2.89 (2.03)	2.30 (1.65)	0.38^b^	0.54 (0.11–0.76)^b^
2-back, F3 latency (ms)	297.29 (47.56)	292.89 (46.84)	0.30	0.47 (−0.03 to 0.72)^b^
2-back, F4 amplitude (μV)	3.37 (2.32)	3.23 (1.89)	0.46^b^	0.63 (0.28–0.81)^a^
2-back, F4 latency (ms)	306.26 (44.99)	308.92 (43.12)	0.09	0.16 (−0.61 to 0.56)

*Abbreviations: ICC, Intraclass correlation coefficient; CI, confidence interval. ^*a*^*p* < 0.0001. ^*b*^*p* < 0.05.*

**FIGURE 2:**
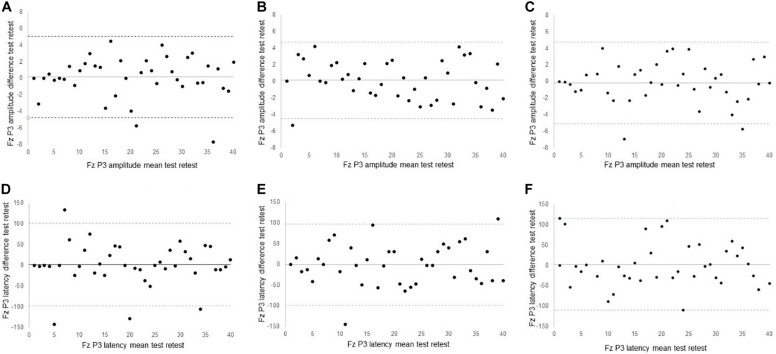
Bland Altman plots of **(A)** 0-back Fz peak amplitude **(B)** 1-back Fz peak amplitude; **(C)** 2-back Fz peak amplitude; **(D)** 0-back Fz peak latency; **(E)** 1-back peak latency; **(F)** 2-back peak latency.

For the main channel location Fz, excellent reliability was found in P3 amplitude for 0-back (ICC = 0.82) and 1-back (ICC = 0.87). P3 amplitude of Fz for 2-back only showed fair reliability (ICC = 0.53). Reliability scores of P3 latency at Fz were fair for 0-back (ICC = 0.54) and 1-back (ICC = 0.47), but poor for 2-back (ICC = 0.17). Figure 2 shows the Bland-Altman plots for P3 peak amplitude and peak latency at the Fz channel. All plots demonstrated equal distribution of the data around zero, indicating no bias in the results and no heteroscedasticity within the data.

Finally, generalized linear mixed models were employed to evaluate the effect of age, disease diagnosis (Aβ−; Aβ+; MCI/AD), cognitive status, and task difficulty on stability of squared P3 peak amplitude and latency at the Fz channel. Age (*p* = 0.74), disease diagnosis (*p* = 0.67), and task difficulty (*p* = 0.70) did not affect the stability of the P3 amplitude response, although individuals with lower MOCA cognitive scores tended to show more variability in P3 amplitude (*p* = 0.07).

Age (*p* = 0.60), disease diagnosis (*p* = 0.55), MOCA (*p* = 0.52), or task difficulty (*p* = 0.95) did not affect the stability of the P3 latency response.

We recalculated ICCs for 0-back, 1-back, and 2-back in participants who scored 26 or higher on MOCA (*n* = 32) and those scoring lower than 26 (*n* = 7). ICC values showed more variance in 0-back and in 2-back in the group with lower MOCA scores, but ICC values were not worse across the *n*-back tests in this group (Supplementary Tables 2 and 3). Whereas ICCs were similar in the Aβ− and Aβ+ groups, lower ICCs were found for the MCI/AD group (Supplementary Tables 2 and 3).

## Discussion

This test-retest reliability study provides critical information on the stability of electrophysiological measures related to working memory in healthy older adults, older adults with increased risk of dementia, and those with MCI or AD. Our results showed that most P3 ERPs in the frontal channels provide fair to excellent reliability to measure electrophysiological processes of cognitive aging in older adults with and without cognitive impairments. Similar to previous studies, the reliability is superior in measures of amplitude compared to latency (Kinoshita et al., 1996; Walhovd and Fjell, 2002; Cassidy et al., 2012; Behforuzi et al., 2019). The robustness of P3 stability is not affected by age, disease diagnosis, or task difficulty, however, there is a trend that lower MOCA scores may affect the stability of the P3 amplitude response.

The body of evidence related to reliability of P3 ERPs is sparse, and typically restricted to healthy young (Segalowitz and Barnes, 1993; Kinoshita et al., 1996; Cassidy et al., 2012; Brunner et al., 2013; Huffmeijer et al., 2014), middle-aged (Kinoshita et al., 1996), and older individuals (Sandman and Patterson, 2000; Walhovd and Fjell, 2002; Behforuzi et al., 2019). Few studies have reported reliability measures in neurological conditions (Lew et al., 2007). The reliability analyses in our study produced fair to excellent ICC values across the *n*-back tests. Whereas ICC values provide a single measure of the magnitude of agreement, Bland-Altman plots depict a graphical display of bias across the two test moments (Ranganathan et al., 2017). Visual inspection of the Bland-Altman plots showed an average difference in ERP responses between first and second testing close to 0, with equal spread of data points around the average difference line. These findings suggest that 2 weeks follow-up is sufficient to wash out any potential adaptation, test, or practice effect of the *n*-back on ERPs in older individuals.

Comparison of our results with other test-retest studies of ERPs in older adults is complicated by lack of consistency in terms of the ERP components that are investigated, the tests of working memory, the choice of channel locations, the extracted P3 metric, the P3 window measurement, and the test-retest reliability intervals (Sandman and Patterson, 2000; Walhovd and Fjell, 2002; Behforuzi et al., 2019). Our research design most closely aligns with a study that compared ERPs to novel stimuli collected at baseline and 7-week follow-up in healthy older individuals (Behforuzi et al., 2019). Similar to our study, this study also found excellent reliability for P3 mean amplitude (ICC = 0.86, 95% CI, 0.78–0.92), and poorer reliability for P3 mean latency (ICC = 0.56, 0.30–0.73). Our study demonstrated larger confidence intervals in some of the amplitude and latency measures, which might have been due to the greater cognitive heterogeneity of our sample. Another study also reported considerably lower reliability in P3 amplitude (ICC = −0.02) and latency (ICC = −0.17) in seven individuals experiencing cognitive difficulties following traumatic brain injury compared to healthy peers (ICC = 0.84 for amplitude and 0.64 for latency) (Lew et al., 2007). Combined, these findings point toward a potential confounding effect of cognitive impairment on stability of ERPs in neurological conditions.

No effect of age, task difficulty, or disease diagnosis was found on stability of the P3 ERP in the *n*-back task. Most participants in our study were cognitively normal, either without (*n* = 15) or with (*n* = 16) elevated Aβ. The fair to excellent reliability of P3 amplitude and latency provides opportunities for studying the effect of Aβ on neural transmission in preclinical AD using ERP. Accumulation of Aβ deposits in the brain is known to increase the risk of developing AD (Klunk et al., 2004). P3 amplitudes are smaller in AD compared to controls (Hedges et al., 2016). ERPs also show useful in predicting conversion to AD, with accuracy rates ranging between 70 and 94% (Chapman et al., 2011). Patients with AD exhibit prolonged latency in P3 ERP compared to age-matched controls (Pedroso et al., 2012). These prolonged latencies observed in patients with AD become particularly apparent in the cognitive domains of executive function, memory, and language (Lee et al., 2013). The ability of P3 ERP to discriminate between MCI and AD (Bennys et al., 2007) opens avenues for investigation of ERP in detecting preclinical AD (Boutros et al., 1995; Rossini et al., 2020).

We established the reliability of P3 amplitude in a group of older adults with a wide range of cognitive ability. Yet, most were cognitive normal. Future studies should include a larger sample of participants with MCI and AD to confirm the confounding effect of impaired cognition on the stability of the P3 response. The results of the group analyses (non β-amyloid elevated; β-amyloid elevated, cognitively impaired), and the potential confounding effect of impaired cognition on ERP response should be considered exploratory. The *n*-back is arguably the most ubiquitous working memory test used in ERP studies across the age spectrum (Bopp and Verhaeghen, 2018). However, previous studies have shown that the *n*-back test hosts an array of control processes, including speed of processing, storage, comparison processes, updating, keeping track, task mixing, task shifting, and resistance to interference (Miller et al., 2009; Schmiedek et al., 2009; Bopp and Verhaeghen, 2018). In addition, we did not establish reliability of ERP in other cognitive domains known to deteriorate in older age, such as memory and language, and this remains an opportunity for further investigation. Future research should also include multiple testing sessions over extended periods of time to evaluate the sensitivity of ERP to detect subtle neurobiological changes due to normal and pathological aging.

## Conclusion

We set out to assess the test-retest reliability of ERP response in older adults with a heterogeneous cognitive profile. Consistent with other studies, P3 amplitude and latency show fair to excellent reliability across different levels of task difficulty. However, impaired cognition may potentially affect the stability of the P3 ERP response.

## Data Availability Statement

The raw data supporting the conclusions of this article will be made available by the authors, without undue reservation.

## Ethics Statement

The studies involving human participants were reviewed and approved by University of Kansas Medical Center Internal Review Board. The patients/participants provided their written informed consent to participate in this study.

## Author Contributions

HD, JB, JM, WB, and KG conceptualized the study. HD, KL, and KG worked out the EEG data processing steps. HD, PA, and KL administered the tests. HD and JM analyzed the data. HD wrote the initial manuscript. JB, KL, PA, JM, WB, and KG reviewed the manuscript and provided valuable comments. All authors contributed to the article and approved the submitted version.

## Conflict of Interest

The authors declare that the research was conducted in the absence of any commercial or financial relationships that could be construed as a potential conflict of interest.
