# Supplementary material for: Reliability of P3 Event-Related Potential During Working Memory Across the Spectrum of Cognitive Aging
Source: Front Aging Neurosci. 2020 Oct 19;12:566391. doi: 10.3389/fnagi.2020.566391 (PMC7604307; doi:10.3389/fnagi.2020.566391)
Supplement: Supplementary file 1 [file Table_1.DOCX]

Supplementary Material

# Supplementary Figures and Tables

## Supplementary Table 1

| Supplementary Table 1. Comparison of performance measures of n-back test at baseline and two-week follow-up (n = 39).^a^ | | | | |
| --- | --- | --- | --- | --- |
| Variable | Baseline | Follow-up | Pearson r | ICC, (95% CI) |
| 0-back, hits | 58.62 (7.28) | 58.62 (6.60) | 0.99 | 0.99 (0.97 - 0.99) |
| 0-back, response time (ms) | 494.68 (103.22) | 519.44 (117.50) | 0.65 | 0.78 (0.59 – 0.89) |
| 1-back, hits | 56.69 (7.30) | 57.03 (7.24) | 0.86 | 0.92 (0.85 – 0.96) |
| 1-back, response time (ms) | 557.55 (116.09) | 555.84 (124.52) | 0.80 | 0.89 (0.79 – 0.94) |
| 2-back, hits | 49.21 (9.81) | 50.69 (9.41) | 0.92 | 0.92 (0.85 – 0.96) |
| 2-back, response time (ms) | 693.89 (146.83) | 698.16 (163.13) | 0.76 | 0.76 (0.58 – 0.86) |
| ICC, intraclass correlation coefficient  ^a^p values of all Pearson r and all ICC were < 0.0001 | | | | |

| Supplementary Table 2. ICCs of P3 peak amplitude at channel location Fz for 0-back, 1-back, and 2-back according to MOCA status and disease diagnosis | | | |
| --- | --- | --- | --- |
| Variable | ICC, (95% CI) | | |
|  | 0-back | 1-back | 2-back |
| MOCA < 26 (n = 7) | 0.76 (-0.16 – 0.92) | 0.95 (0.71 – 0.99)^a^ | 0.58 (-1.45 – 0.16) |
| MOCA ≥ 26 (n = 32) | 0.77 (0.52 – 089.)^a^ | 0.79 (0.57 – 0.90)^a^ | 0.49 (-0.05 – 0.77)^b^ |
|  |  |  |  |
| Aβ- (n = 16) | 0.91 (0.73 – 0.97)^a^ | 0.72 (0.19 – 0.90)^a^ | 0.61 (-0.13 – 0.86)^b^ |
| Aβ+ (n = 17) | 0.78 (0.55 – 0.94)^a^ | 0.91 (0.74 – 0.97)^a^ | 0.54 (-0.17 – 0.77) |
| MCI (n = 7) | 0.60 (-1.02 – 0.92) | 0.93 (0.66 – 0.99)^a^ | 0.63 (-0.84 – 0.93) |
| Abbreviations: ICC, Intraclass correlation coefficient; MOCA, Montreal Cognitive Assessment; Aβ-, cognitive normal older adults with no amyloid elevation; Aβ+, cognitive normal older adults with amyloid elevation; MCI/AD, older adults diagnosed with cognitive impairment  ^a^p < 0.0001; ^b^p < 0.05) | | | |

| Supplementary Table 3. ICCs of P3 peak latency at channel location Fz for 0-back, 1-back, and 2-back according to MOCA status and disease diagnosis | | | |
| --- | --- | --- | --- |
| Variable | ICC, (95% CI) | | |
|  | 0-back | 1-back | 2-back |
| MOCA < 26 (n = 7) | 0.21 (-3.59 – 0.87) | 0.50 (-1.90 – 0.91) | 0.20 (-3.63 – 0.86) |
| MOCA ≥ 26 (n = 32) | 0.57 (0.12 – 0.79)^b^ | 0.56 (-0.03 – 0.79)^b^ | 0.60 (0.12 – 0.79)‑ |
|  |  |  |  |
| Aβ- (n = 16) | 0.60 (-0.15 - 0.86)^b^ | 0.69 (0.10 – 0.89^b^) | 0.61 (-0.13 – 0.86)^b^ |
| Aβ+ (n = 17) | 0.50 (-0.50 – 0.83) | 0.43 (-0.12 – 0.64) | 0.48 (-0.17 – 0.74) |
| MCI (n = 7) | 0.26 (-2.68 – 0.86) | 0.25 (-.2.75 – 0.85) | 0.63 (-0.84 – 0.93) |
| Abbreviations: ICC, Intraclass correlation coefficient; MOCA, Montreal Cognitive Assessment; Aβ-, cognitive normal older adults with no amyloid elevation; Aβ+, cognitive normal older adults with amyloid elevation; MCI/AD, older adults diagnosed with cognitive impairment  ^a^p < 0.0001; ^b^p < 0.05) | | | |
